# Postoperative mortality risk assessment in colorectal cancer: development and validation of a clinical prediction model using data from the Dutch ColoRectal Audit

**DOI:** 10.1093/bjsopen/zrac014

**Published:** 2022-03-31

**Authors:** Lindsey C. F. de Nes, Gerjon Hannink, Jorine ‘t Lam-Boer, Niek Hugen, Rob H. Verhoeven, Johannes H. W. de Wilt, Dutch Colorectal Audit Group

**Affiliations:** 1 Department of Surgery, Maasziekenhuis Pantein, Beugen, The Netherlands; 2 Department of Surgery, Radboud Medical Center, University of Nijmegen, Nijmegen, The Netherlands; 3 Department of Operating Rooms, Radboud Medical Center, University of Nijmegen, Nijmegen, The Netherlands; 4 Department of Surgery, Rijnstate, Arnhem, The Netherlands; 5 Department of Research & Development, Netherlands Comprehensive Cancer Organization, Utrecht, The Netherlands

## Abstract

**Background:**

As the outcome of modern colorectal cancer (CRC) surgery has significantly improved over the years, however, renewed and adequate risk stratification for mortality is important to identify high-risk patients. This population-based study was conducted to analyse postoperative outcomes in patients with CRC and to create a risk model for 30-day mortality.

**Methods:**

Data from the Dutch Colorectal Audit were used to assess differences in postoperative outcomes (30-day mortality, hospital stay, blood transfusion, postoperative complications) in patients with CRC treated from 2009 to 2017. Time trends were analysed. Clinical variables were retrieved (including stage, age, sex, BMI, ASA grade, tumour location, timing, surgical approach) and a prediction model with multivariable regression was computed for 30-day mortality using data from 2009 to 2014. The predictive performance of the model was tested among a validation cohort of patients treated between 2015 and 2017.

**Results:**

The prediction model was obtained using data from 51 484 patients and the validation cohort consisted of 32 926 patients. Trends of decreased length of postoperative hospital stay and blood transfusions were found over the years. In stage I–III, postoperative complications declined from 34.3 per cent to 29.0 per cent (*P* < 0.001) over time, whereas in stage IV complications increased from 35.6 per cent to 39.5 per cent (*P* = 0.010). Mortality decreased in stage I–III from 3.0 per cent to 1.4 per cent (*P* < 0.001) and in stage IV from 7.6 per cent to 2.9 per cent (*P* < 0.001). Eight factors, including stage, age, sex, BMI, ASA grade, tumour location, timing, and surgical approach were included in a 30-day mortality prediction model. The results on the validation cohort documented a concordance C statistic of 0.82 (95 per cent c.i. 0.80 to 0.83) for the prediction model, indicating good discriminative ability.

**Conclusion:**

Postoperative outcome improved in all stages of CRC surgery in the Netherlands. The developed model accurately predicts postoperative mortality risk and is clinically valuable for decision-making.

## Teaser

This Dutch population-based study demonstrates that postoperative complication and mortality rates in colorectal cancer surgery improved in the Netherlands between January 2009 and December 2017. A mortality prediction model was created based on data from a derivation cohort with 51 484 patients and validated in 32 926 patients. The recalibrated validated model has a good calibration and great discriminative ability, and is clinically valuable for decision-making.

## Introduction

The incidence of colorectal cancer (CRC) has increased over the years because of the introduction of national bowel screening programmes and the ageing population^1,2^. Surgical resection of the primary tumour is the cornerstone of curative treatment. CRC surgery is accompanied by postoperative complications and is associated with mortality rates of up to 5 per cent^3^.

Accordingly, accurate individual risk estimation for mortality in CRC surgery could lead to a more patient-tailored approach, improved preoperative counselling, and better outcome by improved decision-making. Factors related to postoperative mortality have previously been evaluated in CRC surgery^4^. Patient- and tumour-related variables such as increasing age, comorbidity, sex, and tumour location in colon or rectum are important factors for postoperative mortality. Previous studies have also demonstrated that patients with stage IV CRC disease develop more postoperative complications and have an increased risk of postoperative mortality than patients with stage I to III disease^4,5^. Multimodal programmes, which focus on improvement of preoperative physical functioning, nutritional intake, and psychological support could potentially enhance recovery and lower postoperative complications and mortality in high-risk patients^6,7^.

Several scoring systems have been developed to assess postoperative mortality risk for patients undergoing CRC resection, but these scoring systems might not represent current clinical practice^8,9^. Postoperative mortality after primary CRC surgery significantly decreased in recent years for both young and elderly patients^10,11^. Most of the models have been developed over 10 years ago in the era prior to laparoscopic surgery and prior to major improvements in perioperative care. As a result, these models regularly overestimate morbidity and mortality risk^12^.

The aim of the present study was to assess nationwide outcomes after CRC surgery and to develop and validate a clinical prediction model for 30-day postoperative mortality using data from a large Dutch nationwide database.

## Methods

Data were derived from the Dutch Colorectal Audit (DCRA), a nationwide multidisciplinary disease-specific initiative. Anony-mized data sets were provided after approval of the research application by the scientific review committee of the Dutch Institute for Clinical Auditing. Data on the tumour, treatment characteristics, and 30-day mortality of surgical patients with CRC are collected by all Dutch hospitals where CRC surgery is performed. Audit participation is obligatory and the data are used for the calculation of quality indicators. Details on the DCRA have been published previously^13,14^. The DCRA only includes postoperative 90-day mortality since 2018 and no data regarding long-term survival. Under Dutch law, for this population study, no informed consent or ethical approval was required.

The present study was performed and reported according to the TRIPOD statement guidelines for the reporting of multivariable prediction models (Supplementary material)^15^.

### Data and variables

ASA Physical Status Classification was used to assess the physical condition of patients at the time of surgery. Classification of tumour characteristics was done according to the TNM Classification of Malignant Tumors and the International Classification of Diseases for Oncology (ICD-O-3). The location of the tumour was divided into right-sided colon (proximal to the splenic flexure), left-sided colon (distal to the splenic flexure; C18–C19), or rectum (C20). Surgical resections were categorized as right colectomy (both ileocaecal resection and right hemicolectomy), transverse resection, left hemicolectomy, sigmoid resection (including anterior resection), (sub)total colectomy, or abdominoperineal resection. Local excisions were excluded.

The timing of surgery was classified as elective, urgent (scheduled with priority, commonly within 2 weeks, e.g. because of impending obstruction), or emergency (unscheduled surgery because of severe complications). Besides the type and classification of surgery, no data were available on the palliative or curative intention of the procedure in patients with stage IV disease. All deaths within 30 days of surgery were registered. Stage IV disease was based on preoperative imaging or was histologically proven before or during surgery. Other variables retrieved and analysed included patient demographics (age and sex), BMI, stoma formation, and additional resections of metastases.

### Outcomes of interest

The primary outcome was 30-day postoperative mortality. Secondary endpoints included blood transfusion, duration of hospital stay, and the occurrence of postoperative complications. Postoperative complications were scored from 2011 as surgical complications when directly attributed to the surgical procedure (e.g. anastomotic leakage) or non-surgical complications when not directly related to the surgery (e.g. postoperative pneumonia). Finally, outcomes included rate of reintervention and days in ICU.

### Statistical analysis and model development

Demographic data, and tumour- and surgery-related information were tabulated. Continuous variables were reported as median (interquartile range (i.q.r.)) or mean (standard deviation) as appropriate. Categorical data were presented as count (percentage).

Trends in postoperative complications, blood transfusion, duration of hospital stay, and mortality of patients with stage I to III disease over time based on data from 2009 to 2017 were tested using χ^2^ tests for trend in proportions or Mann–Kendall trend tests, where appropriate. Trend in surgical complications were based on data from 2011 to 2017 as registration of surgical complications was required from 2011 onwards.

The cohort was divided into two groups: a derivation cohort and a validation cohort. A multivariable prediction model was developed to predict 30-day mortality following primary abdominal resection of CRC, which was subsequently validated.

Patients without (stage I to III disease) and with synchronous metastases (stage IV disease) who underwent CRC resection between January 2009 and December 2014 were included. Patients who underwent only local excision were excluded.

Preoperative patient characteristics that were expected to predict 30-day mortality, based on expert opinion or recent literature, were applied in the initial model. Univariable and multivariable logistic regression modelling were used to test the effect of age, sex, BMI, disease stage (I to III *versus* IV), ASA grade (I, II, III, and IV to V), tumour location (right-sided, left-sided, rectum), timing of surgery (elective, urgent, emergency) and approach (open *versus* laparoscopic). In the final multivariable model variables were selected based on Akaike information criterion^16^. Continuous variables were not dichotomized. Non-linearity of continuous variables was tested with restricted cubic spline (RCS) functions^16^. In the final model restricted cubic spline functions for age and BMI were employed. Data on BMI were missing in 9.1 per cent. As all other variables were more than 99 per cent complete within the derivation cohort, single imputation applying predictive mean matching was used. Internal validation with bootstrap resampling was applied to correct for optimism in the prognostic model. To improve the accuracy of the predictive model, regression coefficients of the model were modified towards zero to reduce overfitting and improve generalizability, using the uniform shrinkage factor correction factor from the bootstrapping. Model performance in the derivation cohort was expressed by discrimination and calibration^16^. Discrimination was quantified by concordance statistic (c), varying between 0.5 for a non-informative model and 1 for a perfectly discriminating model, which refers to the ability to distinguish high-risk patients from low-risk patients. Calibration refers to whether predicted risks agree with the observed outcome, graphically assessed with a flexible calibration plot for the prediction of 30-day mortality, and by calculating a calibration slope and intercept. The calibration slope describes the effect of the predictors in the validation sample *versus* the derivation sample, and is ideally equal to 1. Acalibration intercept is ideally zero and measures if on average the model tends to overestimate or underestimate probability^16,17^. The flexible calibration curve allows examination of calibration across the range of predicted values. A curve close to the diagonal line (i.e. perfect calibration) indicates that predicted (*x*-axis) and observed probabilities (*y*-axis) are corresponding well.

### Validation

For validation, a temporal approach using data from DCRA was used^16^. Patients who underwent primary abdominal CRC surgery for stage I–IV CRC between January 2015 and December 2017 were included. BMI data were missing in 2.6 per cent of the validation cohort. As all other variables were more than 99 per cent complete within the derivation cohort, a single imputation with predictive mean matching was applied.

Following validation, the intercept of the original prediction model was recalibrated. As the incidence of the outcome was lower in the validation set, all predicted risks may be systematically overestimated. In that situation, the intercept (which reflects the risk of the outcome not explained by the covariates) of a prediction model can easily be adjusted, such that the mean predicted risk equals the observed incidence in the validation set. The recalibrated intercept was estimated by fitting a logistic regression model with only an intercept and the linear predictor of the original model as an offset variable (i.e. the coefficient of the linear predictor is fixed at unity)^18^. Model performance in the validation cohort was expressed by discrimination and calibration^16^.

The model was implemented in a web application that provides predictions of 30-day mortality for individual patients undergoing CRC surgery (see below). All statistical analyses were performed with R statistical software (version 4.0.4; R Foundation for Statistical Computing, Vienna, Austria) and the ‘rms’ package (version 6.1-1). The web application was developed with the ‘shiny’ package (version 1.6.0).

## Results

### Baseline characteristics

*Table 1* provides the patient characteristics and perioperative morbidity and mortality data of the derivation (51 484 patients) and validation cohorts (32 926 patients). The patient and tumour characteristics were similar between both groups. However, a large difference was observed between the number of laparoscopic resections that were performed in each cohort: 25 676 patients (49.9 per cent) in the derivation cohort and 25 934 patients (78.8 per cent) in the validation cohort had resections performed laparoscopically.

**Table 1:** Clinical and pathological characteristics of derivation and validation cohorts

	Derivation cohort (2009–2014) (*n* = 51 484)	Validation cohort (2015–2017) (*n* = 32 926)
**Patient characteristics**		
Female sex	22 953 (44.6)	14 389 (43.7)
Mean (s.d.) age (years)	69.76 (11.12)	69.23 (10.32)
Mean (s.d.) BMI	26.10 (4.34)	26.54 (4.62)
ASA grade		
I	10 295 (20.0)	5506 (16.7)
II	29 225 (56.8)	19 430 (59.0)
III	11 109 (21.6)	7451 (22.6)
IV–V	855 (1.7)	539 (1.6)
Preoperative complications	17 052 (33.1)	9070 (27.5)
**Tumour characteristics**		
Clinical stage		
Stage I	12 515 (24.3)	9472 (28.8)
Stage II	17 049 (33.1)	10 208 (31.0)
Stage III	15 917 (31.9)	10 296 (31.3)
Stage IV	5806 (11.3)	2856 (8.7)
Tumour location		
Right	19 202 (37.3)	12 262 (37.2)
Left	17 737 (34.5)	11 397 (34.6)
Rectum	14 545 (28.3)	9267 (28.1)
Morphology		
Adenocarcinoma	46 789 (90.9)	29 243 (88.8)
Mucinous	2574 (5.0)	2529 (7.7)
Signet ring cell	348 (0.7)	291 (0.9)
Other/unknown	1773 (3.4)	863 (2.6)
Differentiation*		
Well/moderate	23 607 (89.3)	27 663 (91.0)
Poor	2821 (10.7)	2737 (9.0)
T-stage†		
pT0	1583 (3.1)	889 (2.7)
pT1	3673 (7.1)	3860 (11.7)
pT2	10 391 (20.2)	7126 (21.6)
pT3	28 991 (56.3)	1680 (51.0)
pT4	6846 (13.3)	4271 (13.0)
N-stage		
pN0	30 631 (59.5)	20 365 (61.9)
pN1	12 634 (24.5)	7894 (24.0)
pN2	7749 (15.1)	4583 (13.9)
pNx	470 (0.9)	84 (0.3)
**Treatment characteristics**		
Timing		
Elective	44 474 (86.4)	29 941 (90.9)
Urgent	3584 (7.0)	1487 (4.5)
Emergency	3426 (6.7)	1498 (4.5)
Surgical resection		
Right-sided	17 055 (33.1)	11 058 (33.6)
Left-sided	4049 (7.9)	2767 (8.4)
Anterior/sigmoid	23 422 (45.5)	15 467 (47.0)
Abdominoperineal resection	4420 (8.6)	2306 (7.0)
Other	2538 (4.9)	1328 (4.0)
Approach		
Open	25 808 (50.1)	6992 (21.2)
Laparoscopic/robotic	25 676 (49.9)	25 934 (78.8)
Stoma		
None	33 669 (65.4)	24 290 (73.8)
Ileostomy	6648 (12.9)	3231 (9.8)
Colostomy	10 688 (20.8)	5363 (16.3)
Stoma – type unknown	479 (0.9)	42 (0.1)
Additional resection metastases (%)	1781 (3.5)	1134 (3.4)
**Outcomes**		
Median (i.q.r.) duration of hospital stay (days)	7.00 (5.00–11.00)	7.00 (5.00–12.00)
Postoperative morbidity	16 694 (32.4)	10 139 (30.8)
Pulmonary	2508 (4.9)	1881 (5.7)
Cardiac	1580 (3.1)	1218 (3.7)
Thromboembolic	316 (0.6)	244 (0.7)
Infectious	1844 (3.6)	1560 (4.7)
Neurological	627 (1.2)	531 (1.6)
Other	3169 (6.2)	3160 (9.6)
Surgical‡	7399 (14.4)	6280 (19.1)
Reintervention	4320 (8.4)	3263 (9.9)
Blood transfusion	7001 (13.6)	3026 (9.2)
ICU admittance		
≤ 2 days	5248 (10.2)	3827 (11.6)
> 2 days	2933 (5.7)	1688 (5.1)
Postoperative mortality		
30-day mortality	1375 (2.7)	535 (1.6)

Data are *n* (%) unless stated otherwise. *Tumour differentiation data were not well registered in the derivation cohort. Missing data were not used for analysis. †This included patients with and without neoadjuvant therapy. ‡Data registration of postoperative surgical complications from 2011 onward. i.q.r., interquartile range.

### Trends of postoperative outcomes

**Fig. 1:**
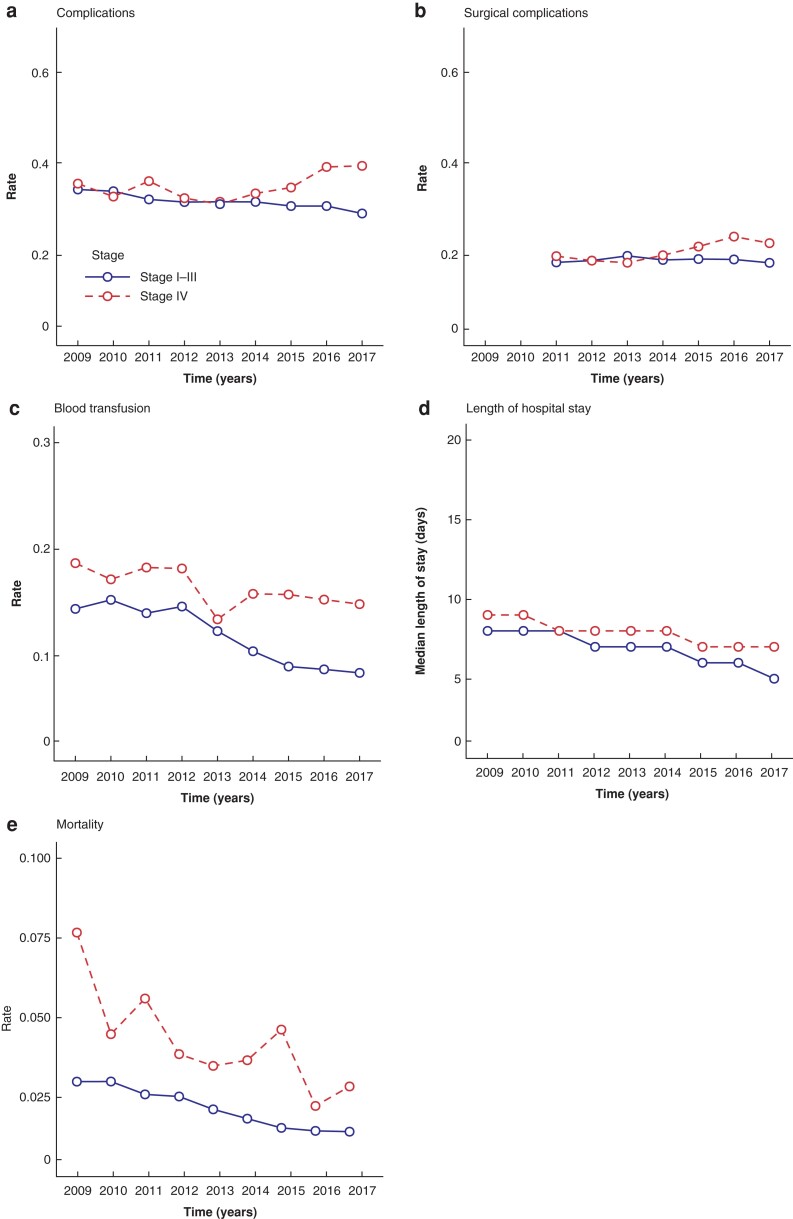
**a** Postoperative complications, **b** postoperative surgical complications, **c** blood transfusion, **d** duration of hospital stay, and **e** postoperative mortality for patients with stage I to III and stage IV colorectal cancer by year. Note that for postoperative surgical complications data registration was required from 2011 onward.

Apparent improvements in postoperative outcome (*Table 1*) during the study period were further explored by trend analyses (*Fig. 1a–e*). In stage I to III disease, analyses showed a lower trend of postoperative complications (from 34.3 per cent to 29.0 per cent; *P* < 0.001) and similar surgical morbidity over time (from 18.3 per cent to 18.2 per cent; *P* = 0.77), whereas increased trends of postoperative (surgical) complications were found in stage IV disease (from 35.6 per cent to 39.5 per cent (*P* = 0.01) and from 19.7 per cent to 22.5 per cent, (*P* = 0.001; Fig. 1a–b)). In both stage I to III and stage IV disease, median duration of hospital stay decreased over time (from 8 days (i.q.r. 6 to 14) to 5 days (i.q.r. 4 to 8) (*P* = 0.002) and from 9 days (i.q.r. 6 to 14) to 7 days (i.q.r. 5 to 12) (*P* = 0.004), respectively; Fig. 1d). Blood transfusion trends lowered from 14.3 per cent to 8.3 per cent (*P* < 0.001) in stage I to III and from 18.6 per cent to 14.8 per cent (*P* = 0.003; Fig. 1c) in stage IV. Mortality declined significantly over the years from 3.0 per cent to 1.4 per cent (*P* < 0.001) in stage I to III disease and from 7.6 per cent to 2.9 per cent in stage IV (*P* < 0.001; Fig. 1e).

### Prediction model

**Fig. 2:**
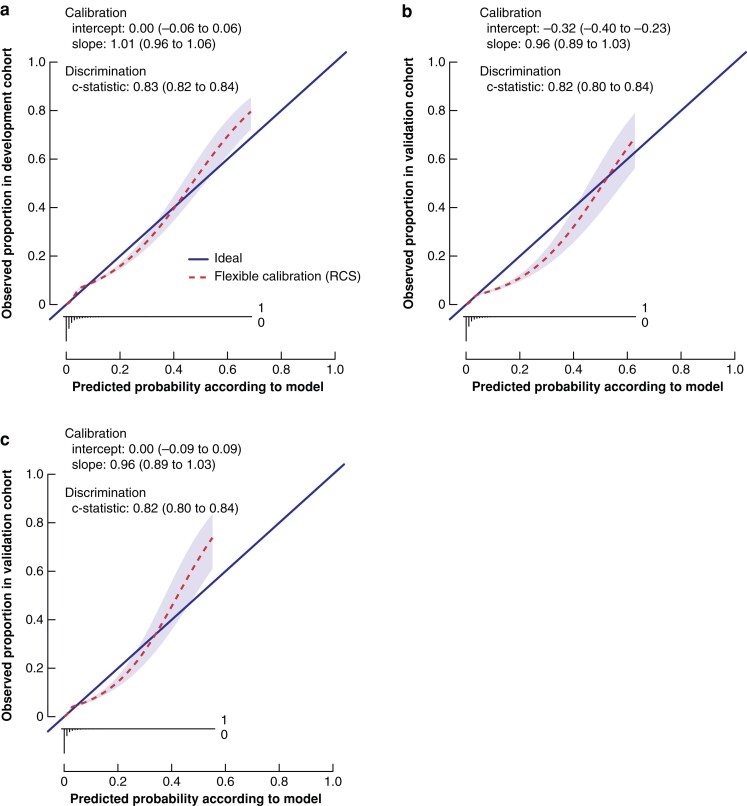
**a** Flexible calibration curve of the original model. Calibration plot for predicted 30-day mortality in the development cohort (51 484 patients). The calibration slope depicts the strength of predictors. The calibration intercept reflects the calibration in the large, indicating whether predicted probabilities are systematically under- or overestimated. The flexible calibration curve allows examination of calibration across a range of predicted values. A curve close to the diagonal line (i.e. perfect calibration) indicates that predicted (*x*-axis) and observed probabilities (*y*-axis) correspond well. The linear bar chart shows the distribution of patients with (= 1) or without (= 0) an observed outcome. Discrimination between a low and high likelihood of 30-day mortality was good (C statistic 0.83, 95 per cent confidence interval (c.i.) 0.82 to 0.84). **b** Flexible calibration curve of the validation model. Calibration plot for predicted 30-day mortality in the development cohort (32 926 patients). Discrimination between low and high likelihood of 30-day mortality was good (C statistic 0.82, 95 per cent c.i. 0.80 to 0.84). The flexible calibration curve shows some overestimation of predicted risks across the range of true risks. **c** Flexible calibration curve of the intercept-adjusted model. Calibration plot for predicted 30-day mortality in the development cohort (32 926 patients). Discrimination between a low and high likelihood of 30-day mortality was good (C statistic 0.82, 95 per cent c.i. 0.80 to 0.84). The flexible calibration curve shows some overestimation of predicted probabilities across the between 5 and 30 per cent range of true risks and some underestimation of predicted probabilities across the range of true risks above 30 per cent. RCS, restricted cubic spline.

All variables that were expected to predict 30-day mortality appeared to be predictors of outcome (*Table 2*). The strongest predictors were ASA grade (odds ratio (o.r.) 3.09, 95 per cent confidence interval (c.i.) 2.17 to 4.40; o.r. 8.16, 95 per cent c.i. 5.72 to 11.63; and o.r. 25.71, 95 per cent c.i. 17.46 to 37.86 (for ASA II, ASA III, and ASA IV to V *versus* ASA I, respectively), age (o.r. 2.78, 95 per cent c.i. 2.46 to 3.13), and non-elective surgery (o.r. 1.67, 95 per cent c.i. 1.40 to 1.98; and o.r. 2.16, 95 per cent c.i. 1.83 to 2.54, for urgent and emergency *versus* elective surgery, respectively). Other variables with a strong association were clinical tumour stage, sex, BMI, tumour location, and approach, which were all included in the final multivariable model. The internally validated C statistic for 30-day mortality was 0.83 (95 per cent c.i. 0.82 to 0.84). Using RCS to model BMI led to deviation from linearity at the lower extreme of the distributions, implying that very low BMI was also associated with higher 30-day mortality risk. The internally validated C statistic for 30-day mortality was 0.83 (95 per cent c.i. 0.82 to 0.84). Using RCS to model BMI led to deviation from linearity at the lower extreme of the distributions, implying that a very low BMI was also associated with higher 30-day mortality risk. The flexible calibration curve, using a restricted cubic spline function, allows examination of calibration across the range of predicted values (*Fig. 2a*). A calibration curve close to the diagonal line indicates that predicted (*x*-axis) and observed probabilities (*y*-axis) correspond well and the deviations of points from the diagonal line with unit slope indicate lack of calibration (see detailed description of calibration steps in *Appendix S1*).

**Table 2:** Predictive accuracy in derivation cohort (*n* = 51 484)

Variables	Univariable model		Multivariable model	
	Odds ratio (95% c.i.)	*P*	Odds ratio (95% c.i.)	*P*
**Stage**				
IIII	1.0		1.0	
IV	1.93 (1.69–2.21)	<0.001	1.75 (1.51–2.04)	<0.001
Age*	3.72 (3.33–4.17)	<0.001	2.78 (2.46–3.13)	<0.001
**Sex**				
Female	1.0		1.0	
Male	1.22 (1.09–1.36)	<0.001	1.45 (1.29–1.64)	<0.001
BMI*	0.83 (0.77–0.89)	<0.001	0.87 (0.81–0.93)	<0.001
**ASA grade**				
I	1.0		1.0	
II	4.99 (3.48–6.99)	<0.001	3.09 (2.17–4.40)	<0.001
III	19.80 (14.02–27.97)	<0.001	8.16 (5.72–11.63)	<0.001
IV–V	81.61 (56.13–118.68)	<0.001	25.71 (17.46–37.86)	<0.001
**Tumour location**				
Right	1.0		1.0	
Left	0.77 (0.68–0.86)	<0.001	0.88 (0.77–1.00)	0.04
Rectum	0.45 (0.38–0.52)	<0.001	0.89 (0.73–1.05)	0.16
**Timing**				
Elective	1.0		1.0	
Urgent	2.82 (2.41–3.31)	<0.001	1.67 (1.40–1.98)	<0.001
Emergency	4.32 (3.76–4.96)	<0.001	2.16 (1.83–2.54)	<0.001
**Approach**				
Laparoscopic	1.0		1.0	
Open	2.63 (2.33–2.96)	<0.001	1.60 (1.40–1.82)	<0.001

* Odds ratios for continuous variables represent interquartile range odds ratios. The presented odds ratios provide insight into the importance of predictors expressed on a relative scale, and can be considered to represent the contribution to the predicted risk. Presented odds ratios do not necessarily represent the causal relation between predictor and outcome or the magnitude of that effect, if any.

### Validation

Similar predictive effects were found for the variables in the validation cohort. The C statistic in the validation cohort was 0.82 (95 per cent c.i. 0.80 to 0.83; *Fig. 2b*).

Calibration in the large depicted a prevalence of 30-day mortality after CRC surgery of 1.6 per cent. The average estimated risk given by the model was 2.2 per cent, indicating that there was a tendency for the model to overestimate risk. Updating the intercept resulted in a decrease in the intercept of 0.32 (see detailed description of model updating and calibration steps in *Appendix S1*). The calibration slope of this updated model was 0.96 (95 per cent c.i. 0.89 to 1.03).

The flexible calibration curve of the final presented validated prediction model with a recalibrated intercept showed some overestimation of predicted probabilities across the 5 to 30 per cent range of true risks and some underestimation of predicted probabilities across the range of true risks above 30 per cent (*Fig. 2c*).

### Web application

The recalibrated validated model was implemented in a web application that provides predictions of 30-day mortality in individual patients undergoing surgery for CRC, for use in clinical practice. It shows the predicted probabilities of 30-day mortality. This web application is accessible at https://crcsurgery.shinyapps.io/predict30daymortality/. Predicted probabilities for individual patients can also be obtained using the information provided in *Appendix S2*.

## Discussion

This large population-based study demonstrated that postoperative outcome in CRC surgery has improved in the Netherlands. Between 2009 and 2017 both duration of hospital stay and the number of postoperative blood transfusions decreased; postoperative mortality decreased from 2.7 per cent to 1.6 per cent for all stages of CRC. Higher ASA grade, older age, and non-elective surgery were the strongest risk factors for postoperative mortality. Tumour stage, sex, BMI, approach (laparoscopic *versus* open), and tumour location were also significant predictors for mortality. A risk model, incorporating these eight clinical baseline characteristics, was developed and validated to predict mortality after CRC surgery on an individual level.

The risk factors for postoperative mortality revealed in the present study have been evaluated in previous studies^19–21^. ASA grade has previously been linked to postoperative mortality in CRC and other gastrointestinal cancers^22–24^. It expresses the operative risk at the moment of surgery and is dependent of variables, such as alcohol or nicotine dependency or ongoing infections and comorbidities. Furthermore, elderly patients had substantially higher risk of death postoperatively, especially octogenarians. An earlier study showed that older patients were more prone to in-hospital mortality^25^. Non-elective CRC procedures increased postoperative complications and mortality in elderly patients, which was most commonly related to frailty^25^. Nevertheless, a previous study demonstrated in 2019 that postoperative mortality decreased significantly in elderly patients with CRC between 2005 and 2016 in the Netherlands^10^.

The association between emergency surgery and postoperative mortality is in agreement with previous reports^20,22,26–28^. The outcome is generally worse as a patient’s condition cannot be optimized preoperatively and dedicated teams are not always available in non-elective settings.

Patients with stage IV CRC have more postoperative complications than those with stage I to III disease, and have an increased risk of postoperative mortality^4,5^. In contrast to stage I to III CRC, only a subset of patients with stage IV disease undergoes CRC surgery. A recent meta-analysis demonstrated that relatively younger and healthier patients with metastatic CRC were more likely to undergo surgery and that short-term mortality was reduced in resected patients, resulting in a modest positive effect on survival^29^.

In this study, both high and low BMI were associated with a higher postoperative mortality risk in patients with CRC. In accordance with this finding, a multicentre observational cohort study of 11 995 patients with rectal cancer who underwent proctectomy showed an increased association with postoperative mortality and sepsis in patients with la ow BMI^19^. A low BMI might reflect poor nutritional state, less healthy physiology, or it could be caused by more advanced disease^19^. In a relatively small regional study using data from the Netherlands cancer registry, underweight patients with CRC not only had worse short-term outcome than normal-weighted patients, but it also led to worse long-term survival^21^.

Several risk scores exist to predict long- and short-term outcomes of CRC surgery^30,31^. The predictive performance of these prediction models deteriorate over time, a phenomenon called ‘calibration drift’^16^. These models are generally based on retrospective series with high postoperative mortality and, as a result, overestimate postoperative mortality^12^. The development of historical models took place in the era prior to laparoscopic surgery and major improvements in perioperative care. As a consequence, the use of these tools could lead to inadequate patient counselling. The model presented in this study is up to date, based on modern high-quality data, and will therefore estimate postoperative mortality more accurately. An important advantage of the present model is that it relies on prospectively gathered, mostly continuous data from a large, unselected national cohort. This is different from previous models, which are usually based on retrospective data^8,9^. Improvements in perioperative CRC surgery, such as laparoscopy, centralization of care, and specialization and implementation of enhanced recovery after surgery (ERAS) care, have probably contributed to the reduction in postoperative complications and mortality reported in the present study^19,29–32^. These improvements reflected in the trend analysis were considered in the final model; otherwise, the predicted risks in new patients would be systematically too high. For this, a recalibration method (i.e. adjustment of the intercept of the prediction model) was used to improve the model’s calibration and to provide more accurate predictions in the new patient population. With this adjustment, the present model has a good calibration and great discriminative ability.

Model updating is crucial, as the use of deteriorated risk models may lead to over- or underestimation of a patient’s surgical risk and incorrect benchmarking results. Therefore, this risk prediction model needs to be periodically updated^16^. The traditional regression approach was used instead of modern machine-learning techniques. With these methods automatic recalibration is feasible and possibly unknown postoperative risk factors could be identified^16^. However, a recent systematic review showed no performance benefit of machine learning over logistic regression for clinical prediction models^32^. This is also confirmed by the present results, and the model based on a traditional logistic regression approach (area under the curve (AUC) 0.82, 95 per cent c.i. 0.80 to 0.83) performed equally well (AUC 0.82, 95 per cent c.i. 0.80 to 0.83) as the best-performing machine-learning model for 30-day mortality in CRC (AUC 0.82, 95 per cent c.i. 0.79 to 0.85)^33^.

The data used in the present study were derived from the DCRA database^34^. Population-based data reflect daily practice without patient selection, which is in contrast to clinical studies or single-centre studies, in which selection bias could occur. Nevertheless, some limitations apply to the data from the DCRA. Firstly, some essential data are not available in the DCRA, such as vital parameters and biochemical values (e.g. haemoglobin, albumin, and urea), which have been demonstrated to be important in previous studies^8^. Also, information on steroid use, frailty, socio-economic status, deprivation, volume of cases per unit, or on enhanced recovery programmes was not available. The possible impact of not including certain parameters is not completely clear, but does appear to depend on the strength of the (unavailable) predictor(s). For example, as the discriminative ability of a prediction model will largely be based on the strongest predictors, the unavailability of a strong predictor tends to lead to a lower c-index. In addition, ignoring a strong predictor will cause all predicted risks to be too low and results in a worse calibration. The calibration slope will also be affected, as the predicted probabilities become more alike (less extreme) owing to the unavailability of a strong predictor. However, if the predictors in the model have a predictive strength similar to the unavailable predictor(s) the model may lead to a similar or even better performance^35^. Secondly, the details of specific comorbidities were not available for further analysis, but ASA grade also considers comorbidities. Furthermore, details regarding lifestyle factors, such as diet, smoking, and physical condition have been demonstrated to be important factors in postoperative outcome^36,37^. These factors are not scored in the DCRA but have recently received increased attention in the literature^6,7^. In particular, frail patients and those with impaired performance status, considered to be at high risk of postoperative complications, might profit from personalized multimodal prehabilitation. The model in the current study could be used to identify patients at increased risk of postoperative mortality; potentially, these patients could be introduced in such prehabilitation programmes in order to improve their condition.

Lastly, whether the intention of the resection was curative or palliative, and specifications on the type of additional metastasectomy, were unknown. Furthermore, the location and number of organs affected by metastases in patients with stage IV disease could not be extracted from the database and, as a result, their additional value in postoperative mortality risk could not be investigated. As a result of these lacking data, the chance exits of selecting relatively healthy patients with stage IV disease being treated with curative intent. Further, the developed risk model could help in guiding the challenging task of selecting patients with stage IV disease who might benefit most from colorectal surgery.

## Supplementary Material

zrac014_Supplementary_Data

## Data Availability

The authors agree to make data, analytic methods, and study materials available to other researchers on request.
