# Supplementary material for: Postoperative mortality risk assessment in colorectal cancer: development and validation of a clinical prediction model using data from the Dutch ColoRectal Audit
Source: BJS Open. 2022 Mar 31;6(2):zrac014. doi: 10.1093/bjsopen/zrac014 (PMC8969795; doi:10.1093/bjsopen/zrac014)
Supplement: zrac014_Supplementary_Data [file zrac014_supplementary_data.zip › Supplementary_Appendix_1.docx]

**Appendix S1.** Detailed illustration of the assessment of calibration and model updating (recalibration)

1. **Development and internal validation**

The model was developed and internally validated to predict the probability of death within 30 days after CRC surgery using data from 51,484 adult patients who underwent primary abdominal CRC surgery for stage I-IV colorectal cancer between January 2009 and December 2014. Data were derived from the Dutch Colorectal Audit (DCRA), a nationwide multidisciplinary disease-specific initiative. The model uses eight predictors: Stage (I-III, IV), Age, Sex (female, male), BMI, ASA (I, II, III, IV-V), Tumor location (right-sided, left-sided, rectum), Timing (elective, urgent, emergency), and Approach (open, laparoscopic).

See figure 2a for a flexible calibration curve of the original (internally validated) model and info on calibration intercept and calibration slope and discrimination.

The model after internal validation is given as:

LP = - 7.717855

+ 0.5561493 * [Stage: IV]

+ 0.05188826 * Age + 0.00002137224 * (Age -55)^3^_#_

- 0.00004986857 * (Age - 71)^3^_#_ + 0.00002849632 * (Age - 83)^3^_#_

- 0.3719148 * [Sex: Female]

- 0.07249994 *BMI + 0.0007049504 * (BMI - 21.19274)^3^_#_

- 0.001226326 * (BMI - 25.60554)^3^_#_ + 0.0005213759 * (BMI - 31.57207)^3^_#_

+ 1.116276 * [ASA2] + 2.077444 * [ASA3] + 3.213088 * [ASA4/5]

- 0.1295814 * [Tumor location: Left] - 0.1149924 * [Tumor location: Rectum]

+ 0.5048523 * [Timing: Urgent] + 0.7620514 * [Timing: Emergency]

+ 0.464099 * [Approach: Open]

and [c] = 1 if subject is in group c, 0 otherwise; (x)_#_ = x if x > 0, 0 otherwise.

LP = linear predictor; the linear predictor is the weighted sum of the values of the predictors in the model, where the weights are the regression coefficients.

The probability (P) of death within 30 days after CRC surgery is then obtained as follows:

*P*_30-day mortality_ = $\frac{\text{1 }}{\text{1+exp(-LP)}}$

First, the external predictive performance of the model was evaluated on data from 32,926 adult patients who underwent primary abdominal CRC surgery for stage I-IV colorectal cancer between January 2015 and December 2017 using steps and terminology used in van Calster et al.^1^ Data were derived from the Dutch Colorectal Audit (DCRA), a nationwide multidisciplinary disease-specific initiative.

1. *Van Calster B, McLernon DJ, van Smeden M, Wynants L, Steyerberg EW; Topic Group ‘Evaluating diagnostic tests and prediction models’ of the STRATOS initiative. Calibration: the Achilles heel of predictive analytics. BMC Med 2019;17(1):230*

As a second step the model was updated.

1. **External validation**
   1. Discrimination

The area under the ROC curve (AUC) was estimated at 0.82 (95% CI 0.80–0.84), which suggests good discrimination between patients who die within 30 days after CRC surgery and patients who do not die within 30 days after CRC surgery in our data.

- 1. Mean calibration (calibration-in-the-large)

The prevalence of 30-day mortality after CRC surgery was 1.6%. The average estimated risk given by the model was 2.2%, which indicates that there was a tendency to give overestimated risks by our model.

- 1. Calibration intercept and calibration slope

To estimate the calibration intercept, the logistic model Y = α + LP is fitted. In logistic regression, Y is the logarithm of the estimated risk (*P*_30-day mortality_) divided by 1 minus the estimated risk, i.e., log(*P*_30-day mortality_ /(1– *P*_30-day mortality_)). Notice that there is no regression coefficient for the effect of LP, which is equivalent to setting the coefficient of LP to 1. In a practical sense, this means that a regression model is fitted with LP as an ‘offset term’. The estimated value of the intercept α is the calibration intercept.

To obtain the calibration slope, the logistic model Y = α’ + ß*LP is fitted. The estimated value of the slope ß is the calibration slope.

In the present dataset, the calibration intercept was -0.32 (95% CI -0.40– -0.23), with zero as the target value, suggesting the tendency to give overestimated risks. The calibration slope was 0.96 (95% CI 0.89–1.03), which is very close to the target value of 1. This calibration slope suggests that risk estimates were not systematically too extreme or moderate.

- 1. Flexible calibration curve

The flexible calibration curve is based on the logistic model Y = α” + *f*(LP).  *f* is a flexible smoothed continuous function based on loess or spline functions, for example. In the example, a restricted cubic spline curve was used. This curve showed an overestimation of predicted risks across the range of true risks (Fig. 2b). The flexible calibration curve for the original (internally validated) model is presented in Fig. 2a. The calibration curves were generated using the `val.prob.ci.2` function from the Calibration Curves package for R (<https://github.com/BavoDC/CalibrationCurves>). R version 4.0.4 was used ([www.R-project.org](http://www.R-project.org)).

1. **Updating of a logistic regression model**
   1. Updating using intercept adjustment (recalibration)

Intercept adjustment is the simplest form of model updating. Of the model’s original regression coefficients, only the intercept is re-estimated. Updating can be considered when the calibration intercept is not close to 0, as in our example. Adjusting the intercept is done by adding the calibration intercept (-0.316368) to the model intercept (- 7.717855). The new, updated LP of the model then becomes:

LP_recal_ = - 8.034223

+ 0.5561493 * [Stage: IV]

+ 0.05188826 * Age + 0.00002137224 * (Age -55)^3^_#_

- 0.00004986857 * (Age - 71)^3^_#_ + 0.00002849632 * (Age - 83)^3^_#_

- 0.3719148 * [Sex: Female]

- 0.07249994 *BMI + 0.0007049504 * (BMI - 21.19274)^3^_#_

- 0.001226326 * (BMI - 25.60554)^3^_#_ + 0.0005213759 * (BMI - 31.57207)^3^_#_

+ 1.116276 * [ASA2] + 2.077444 * [ASA3] + 3.213088 * [ASA4/5]

- 0.1295814 * [Tumor location: Left] - 0.1149924 * [Tumor location: Rectum]

+ 0.5048523 * [Timing: Urgent] + 0.7620514 * [Timing: Emergency]

+ 0.464099 * [Approach: Open]

and [c] = 1 if subject is in group c, 0 otherwise; (x)_#_ = x if x > 0, 0 otherwise.

After this intercept update, the flexible calibration curve of the intercept-adjusted model is closer to the diagonal reference line of perfect moderate calibration (Fig. 2c), however, the curve shows some overestimation of predicted probabilities across the between 5 and 30% range of true risks and some underestimation of predicted probabilities across the range of true risks above 30%.
