# Supplementary material for: Postoperative mortality risk assessment in colorectal cancer: development and validation of a clinical prediction model using data from the Dutch ColoRectal Audit
Source: BJS Open. 2022 Mar 31;6(2):zrac014. doi: 10.1093/bjsopen/zrac014 (PMC8969795; doi:10.1093/bjsopen/zrac014)
Supplement: zrac014_Supplementary_Data [file zrac014_supplementary_data.zip › Supplementary_Appendix_2.docx]

**Appendix S2.** Manual calculation of predicted risk for an individual patient

The probability (*P*) of death within 30 days after CRC surgery can be calculated using the following formula:

*P*_30-day mortality_ = $\frac{\text{1 }}{\text{1+exp(-lp)}}$, where

lp = - 8.034223

+ 0.5561493 * [Stage: IV]

+ 0.05188826 * Age + 0.00002137224 * (Age -55)^3^_#_

- 0.00004986857 * (Age - 71)^3^_#_ + 0.00002849632 * (Age - 83)^3^_#_

- 0.3719148 * [Sex: Female]

- 0.07249994 *BMI + 0.0007049504 * (BMI - 21.19274)^3^_#_

- 0.001226326 * (BMI - 25.60554)^3^_#_ + 0.0005213759 * (BMI - 31.57207)^3^_#_

+ 1.116276 * [ASA2] + 2.077444 * [ASA3] + 3.213088 * [ASA4/5]

- 0.1295814 * [Tumor location: Left] - 0.1149924 * [Tumor location: Rectum]

+ 0.5048523 * [Timing: Urgent] + 0.7620514 * [Timing: Emergency]

+ 0.464099 * [Approach: Open]

and [c] = 1 if subject is in group c, 0 otherwise; (x)_#_ = x if x > 0, 0 otherwise.

For example, a 79-years-old female patient with stage III, right-sided tumor, ASA3, BMI of 25 kg/m^2^, timing of surgery urgent, and operated using an open approach would have probability of death within 30 days after CRC surgery of ~6%, see calculation below.

lp = - 8.034223

+ 0.5561493 * 0

+ 0.05188826 * 79 + 0.00002137224 * (24)^3^_#_

- 0.00004986857 * (8)^3^_#_ + 0.00002849632 * (0)^3^_#_

- 0.3719148 * 1

- 0.07249994 * 25 + 0.0007049504 * (3.80726)^3^_#_

- 0.001226326 * (0)^3^_#_ + 0.0005213759 * (0)^3^_#_

+ 1.116276 * 0 + 2.077444 * 1 + 3.213088 * 0

- 0.1295814 * 0 - 0.1149924 * 0

+ 0.5048523 * 1 + 0.7620514 * 0

+ 0.464099 * 1 = -2.764247

*P*_30-day mortality_ = $\frac{\text{1 }}{\text{1+exp(- -2.764247)}}$ = 0.05928705
